# Supplementary material for: Characterization of the Burkholderia cenocepacia J2315 Surface-Exposed Immunoproteome
Source: Vaccines (Basel). 2020 Sep 6;8(3):509. doi: 10.3390/vaccines8030509 (PMC7565204; doi:10.3390/vaccines8030509)
Supplement: Supplementary file 1 [file vaccines-08-00509-s001.zip › S4.pdf]

Table S4. Predicted surface-exposed proteins identified in *B. cenocepacia* J2315 by using the "surface shaving" strategy followed by LC-MS/MS (Threshold score: ≥5 peptides) and their predicted B cell epitopes.

ORF	Description	Peptides identified [1]	Predicted B cell epitopes [2]	B cell epitopes average (predicted peptides) [2]
BCAL0304	VacJ-like lipoprotein	^{46}FNDTVDTYALKPVAK^{60}<br>^{96}IADGVGDIMR^{105}<br>^{195}ANLLGAGDVLDAAALDK^{211}<br>^{21}7NAYLQR^{222}<br>^{232}GEAAVTSNNDALPK^{245}	^{27}VQTPTKGDPLE^{37}<br>^{125}KLPKHTADF^{133}<br>^{175}YVKPDG^{180}<br>^{194}R**ANLLGAGDV**^{203}	0.469 (4)
BCAL0342	Type VI secretion system protein TssC	^{100}GLDYLVK^{106}<br>^{132}GASEFDQSALFK^{143}<br>^{237}YVGLTLPR^{244}<br>^{304}GVEGGGLVEDLPTHTFK^{320}<br>^{486}LVADLPKPANS^{496}	^{5}TAAAQASGAEYAAGTSL^{21}<br>^{27}EKSKVAKSDSEHAR^{40}<br>^{54}DGTVIVSDN^{62}<br>^{90}EFQRLES^{96}<br>^{109}NTGQT^{113}<br>^{127}VRDFK**GASEFDQSAL**^{141}<br>^{143}**K**KVYEEEFGTFGG^{155}<br>^{167}ISRQPED^{173}<br>^{250}PFNPKDGQTAENFNFVEDVDGTDH^{273}<br>^{301}AIR**GVEGGGLVED**^{313}<br>^{320}**K**TDDGEVALKCP^{331}<br>^{337}TDRREKE^{343}<br>^{371}KPKKYSTDSANA^{382}<br>^{411}IGSFASAQNVET^{422}<br>^{431}YVLLDDNASQEQKAQFPLR^{449}	0.472 (15)
BCAL1288	Family M23 peptidase	^{56}WNQMTNPDAIEVGQVLR^{72}<br>^{93}ARPAPSAPVESAVKPATSIALIWPAAGNVV^{122}<br>^{132}GIDIANSPGTPVVAAAPGVVVYAGNGLR^{159}<br>^{160}GYGNLIILK^{168}<br>^{187}EGQSVTQGQSIAEMGNSDSDR^{207}<br>^{208}VALHFELR^{215}	^{17}LLVACGSAPVGP^{28}<br>^{121}**VV**RTFDGSK^{129}<br>^{182}ALLVK**EGQSVTQ**^{193}<br>^{202}**NSDSDR**^{207}<br>^{219}RSIDP^{223}	0.513 (5)
BCAL1881	Putative lipoprotein	^{59}YLFSPVAVGDAVYAAGENGSVEK^{81}<br>^{95}VGSDLSAGVGSDGNLTAVGALK^{116}	^{25}SSKDARRVPTPLTEFKPVMDV^{45}<br>^{51}ASVGKGG^{57}	0.444 (8)

		^{117}GGVFVLGPDGK^{127}<br>^{153}TIDGQVIAFNAQTGEQK^{169}<br>^{350}TDGSAITAAPVLAGNTLVVQTK^{371}	^{91}WRSK**VGS**^{97}<br>^{127}**K**QLWKTSVQ^{135}<br>^{167}**EQK**WNYRNRAVPLNLRV^{183}<br>^{217}QTPVSFPKGVTEVERIND^{234}<br>^{263}GRPLWEKPFSSRSGLAQ^{279}<br>^{302}NQLWRNDKLKSR^{313}	
BCAL1893	Family M23 peptidase	^{51}SGSLGTTATAQPAVPLGPPPPGFYR^{75}<br>^{95}DIASWNNLANPNQIEVDQLLR^{115}<br>^{188}GPVLNGFDDAK^{198}<br>^{199}NKGVNIGGTAGEAVK^{213}<br>^{220}VVYSGNGLR^{228}<br>^{238}HDATYLTAYAHNR^{250}<br>^{266}IAEMGNSDADR^{276}<br>^{286}QGKPVDPLK^{294}	^{5}SMLRAMQNNRSREPLTLA^{22}<br>^{191}**LNGFDDAK**^{198}<br>^{251}ALMVKEGDAVTK^{262}<br>^{287}**GKPVDPLK**Y^{295}	0.523 (4)
BCAL1985	Putative exported isomerase	^{38}ADAMVAQLVQQGQTDGPQLQQAVR^{61}<br>^{68}EILMQEAIR^{76}<br>^{87}AQVAVAQQTVVLR^{99}<br>^{186}AYVPEFAAAAQK^{197}<br>^{235}AQIAQQLVQQK^{245}<br>^{246}LQAFEEGLR^{254}	^{45}**LVQQGQTDGPQLQQAVR**QELVNR**E**^{68}<br>^{78}GIPNRPD^{84}<br>^{198}LQKGQMTDT^{206}<br>^{221}DIRDIAPPPF^{230}<br>^{240}**QLVQQKLQAFEEG**^{252}	0.494 (5)
BCAL2022	PspA/IM30 family protein	^{13}GLLNDAADSVQDPSR^{27}<br>^{35}ELDDSIGR^{42}<br>^{43}AENSLIEIEAQVATQR^{58}<br>^{78}ALQGGDEALAR^{88}<br>^{89}EALAAQSNAEAER^{101}<br>^{150}DVAASALGGIGGK^{162}	^{20}**DSVQDPSRD**^{28}<br>^{156}**LGGIGGK**NLSEDFQKLEDK^{174}<br>^{215}AALKKQLD^{222}	0.503 (3)
BCAL2083	Outer membrane protein assembly factor YaeT	^{44}VEAGSVFAYLPIK^{56}<br>^{111}EFDKDNLTK^{119}<br>^{152}GFYAAEVK^{159}<br>^{226}LTGDLEAVR^{234}<br>^{319}LGEYGYAFATVNAQPDIDQANHK^{341}<br>^{342}VNLNLVVDPSR^{352}	^{41}LQR**VEAGSV**^{49}<br>^{54}**PIK**QGDTFTDDKAS^{67}<br>^{111}**EFDKDNL**^{117}<br>^{124}VGLADGRYYDKALVDKAEQ^{142}<br>^{192}NKAFSTSTLRDE^{203}<br>^{360}NVVGNTRTRDEV^{371}<br>^{373}RREMRQLESSWFDSNRL^{389}	0.488 (19)

			^{410}VPVDGTN^{416}<br>^{426}DEKPTGA^{432}<br>^{454}DNVFGSGT^{461}<br>^{484}YFTVDGIKR^{492}<br>^{498}YRTYQPLYYSTSSSFR^{513}<br>^{574}WSRDARDSALIPSRG^{588}<br>^{595}IEYGVPVGKIQ^{605}<br>^{611}LQAQYYYSFS^{620}<br>^{634}GNGIGNPYP^{642}<br>^{657}GYEPSSLGPRDTKTNDPI^{674}<br>^{712}GNAPGGTSTGANG^{724}<br>^{751}QKHEGDQYQ^{759}	
BCAL2645	OmpA family protein	^{79}LAPSAAQTGTQVTEQPDGSLK^{99}<br>^{161}AQSVVNALVQR^{171}<br>^{178}LSAQGMGASNPIADNATEAGR^{198}<br>^{203}RVEIYLR^{209}<br>^{204}VEIYLR^{209}	^{88}**TQVTEQ**^{93}<br>^{109}ATNQYAITPA^{118}<br>^{145}DSTGSAQLNQTL^{156}<br>^{183}**MGASNPIADNATEAGR**AQN^{201}	0.502 (4)
BCAL2958	OmpA family protein	^{93}ITYQADALFDFDK^{105}<br>^{93}ITYQADALFDFDKATLKPLGK^{113}<br>^{114}QKLDELASK^{122}<br>^{116}LDELASK^{122}<br>^{123}IEGMNTEVVVATGYTDR^{139}<br>^{203}RVEVEVVGTQQVQK^{216}<br>^{204}VEVEVVGTQQVQK^{216}	^{101}**FDFDKATLKPLGKQ**^{114}<br>^{139}**R**IGSDKYNDRL^{149}<br>^{211}**TQQVQK**TTV^{219}	0.512 (3)
BCAL3204	Putative OmpA family lipoprotein	^{31}ANNAGAVSTQPSADNVAQVNVDPLNDPNSPLAK^{63}<br>^{65}SIYFDFDSYSVK^{76}<br>^{97}HVLIQGNTDER^{107}<br>^{108}GTSEYNLALGQK^{119}<br>^{127}AMALLGVNDSQMEAVSLGK^{145}<br>^{146}EKPQATGHDEASWAQNR^{162}	^{67}**YFDFDSYSVK**DEYQP^{81}<br>^{105}**DERGTSEYNLAL**^{116}<br>^{143}**LGKEKPQATGHDEASWAQN**^{161}	0.507 (3)
BCAL3311	BcnA	^{41}QMNVPTEGAFKK^{52}<br>^{41}QMNVPTEGAFK^{51}<br>^{64}AAQGSAQMTIDVASFDLGDK^{83}	^{21}SGAAHADV^{28}<br>^{78}**FDLGDKMYNDQVAGKDWFDAK**^{98}<br>^{158}**AFNVGTGEWKDTSIVA**^{173}	0.455 (3)

		^{84}MYNDQVAGK^{92}<br>^{93}DWFDAK^{98}<br>^{99}TYPQATFVSSAIAPAGGNK^{117}<br>^{128}GKAETVTVPVTVAQNGATQTFDGVLPIK^{155}<br>^{128}GKAETVTVPVTVAQNGATQTFDGVLPIKR^{156}<br>^{130}AETVTVPVTVAQNGATQTFDGVLPIK^{155}<br>^{157}SAFNVGTGEWK^{167}<br>^{168}DTSIVADEVQIK^{179}<br>^{180}FHLVATK^{186}		
BCAM1931	Putative porin	^{44}SLWSMGSGIDQSR^{56}<br>^{61}GSEDLGGGLK^{70}<br>^{86}LGNNGGMFNR^{95}<br>^{96}QAFVGLSSQYGTVTLGK^{112}<br>^{144}LSTNGDVALNNSIK^{157}<br>^{185}AYSGGLSYQFQGLK^{198}<br>^{199}LGAAYSQANLGDGTNANGATNIAAQGR^{225}<br>^{263}ADNYEVNAK^{271}<br>^{272}YNLTPALGLGAAYTYTNAK^{290}<br>^{313}TDVYAQAVYQR^{323}<br>^{327}NAGGASIYNGDLSTAPSSSINQTAATVGLR^{356}	^{36}YQSNVASK**SLWSMGSGID**^{53}<br>^{63}**EDLGGG**^{68}<br>^{79}FNIGNGR**LGNNGGMF**^{93}<br>^{173}SFSNNQNFGNNR**A**^{185}<br>^{192}**YQFQG**^{196}<br>^{205}**QANLGDGTNANGATNIAAQGR**V^{226}<br>^{248}QSRIDNRAAGVPSLR**ADN**^{265}<br>^{272}**YNLTPA**^{277}<br>^{287}**TNAK**VDGSSAHW^{298}<br>^{323}**R**ASK**NAGGASIYNGDLSTAPSSSI**^{346}	0.503 (10)
BCAM2549	Multidrug efflux system outer membrane protein OpcM	^{162}AQGTVSYEADLFGR^{175}<br>^{185}ADQAQSEALFR^{195}<br>^{240}FNEGDISELDVSR^{252}<br>^{255}NELASAQADAVGVAR^{269}<br>^{396}AQYDEQVANYR^{406}	^{4}DRKMDNMHNTNGLMR^{18}<br>^{175}**R**VGRNVEASR**ADQAQSEAL**^{193}<br>^{264}**AVGVAR**RRAA^{273}<br>^{286}PADFAFKETPIVPVAVKIPPGL^{307}<br>^{316}PDVSA^{320}<br>^{332}IGLAKSAYFPK^{342}<br>^{346}TGSFGYEASTLGNLFLWSSRTFLLG^{370}<br>^{421}ADLRLLDDQIRAQEAAVNASRRAAT^{445}<br>^{448}RTQYQEGEVAYL^{459}<br>^{466}RSVLQSQLQANQ^{477}<br>^{495}GGWGNAPAPTAVGDAASGKAD^{515}	0.521 (11)
BCAM2761	Giant cable pilus	^{80}LATAPALKNQTSPGAAEIPLSVK^{102}<br>^{88}NQTSPGAAEIPLSVK^{102}	^{67}IFTNDKAKD^{75}<br>^{85}ALK**NQTSPGAA**^{95}	0.501 (2)

^{103}LGETELTTTAATLK^{116}
^{117}TAELFPGELAQGSNVLALSIGQK^{139}
^{140}KVEAVTASGSYQGLVSVIVTQSAASGS^{166}

[1] Peptides identified using surface-shaving with trypsin and LC-MS/MS analysis. [2] http://tools.iedb.org/bcell/. Threshold used of 0.5. Peptides shorter than 5 or larger than 25 amino acids were not considered. At bold are the predicted B cell epitopes found by the Surface-shaving approach.
